# Bowel perforation after liposuction in abdominal contouring surgery: Case report

**DOI:** 10.1016/j.ijscr.2020.05.012

**Published:** 2020-05-21

**Authors:** Caterina Gardener, Laura Pandis, Martina Grigatti, Vincenzo Vindigni, Franco Bassetto, Tito Brambullo

**Affiliations:** Clinic of Plastic and Reconstructive Surgery, Neuroscience Department, University of Padua, Italy

**Keywords:** Intestinal perforation, Flank hernia, Body contouring, Liposuction, Case report, Combined surgery

## Abstract

**Introduction:**

Liposuction is one of the most performed procedures in aesthetic plastic surgery worldwide, and its spectrum of applications covers almost all body areas.

Systemic or visceral complications following liposuction are rare, but unfortunately, they can be very serious.

**Presentation of case:**

We discuss the case of a 69 y.o. woman who underwent abdominal contouring surgery consisting in flank pseudohernia correction, liposuction and short scar abdominoplasty, which was complicated by intestinal perforation.

Bowel perforation was suspected on the 3rd day following surgery after a CT-scan and was treated with exploration through a median laparotomy, resection of the perforated bowel and subsequent ileo-ileal anastomosis. The 10 cm-long resected segment of the small intestine presented multiple 2 mm-large holes at the microscopic examination.

**Discussion:**

We assume that patient position on the operating table and abdominal wall laxity during surgery as well as the timing of each specific procedure played an important role in the occurrence of bowel perforation.

**Conclusion:**

To our knowledge, this is the first case report of an accidental visceral perforation during a combined procedure of flank bulging correction and abdominal liposuction.

Overall the increased risk of combined procedures in plastic surgery is linked to increased operative time. In the current case timing of surgery sequence more than operative time itself was relevant in increasing anterior abdominal wall pressure and thus setting the patient at a higher risk of bowel perforation.

## Highlights

• To our knowledge, this is the first case report of an accidental visceral perforation during a combined procedure of flank bulging correction and abdominal liposuction.
• Complications following liposuction are rare, but they can be very serious, such as penetration of the abdominal wall and consequent lesion of one or more viscera.
• Patient position on operating table and abdominal wall laxity during surgery as well as the timing of each specific procedure played an important role in the occurrence of bowel perforation.

## 1 Introduction

Nowadays literature reports a low incidence of both local and systemic complications of liposuction, among the latter the most severe include: deep venous thrombosis, pulmonary embolism, cavities perforation, necrotizing fasciitis, sepsis and heart attack [1].

Penetration of the abdominal wall and consequent lesion of one or more viscera is rare and underestimated, but it represents a life-threatening complication. Awareness of this kind of complication is essential to promptly treat the patient and avoid dramatic consequences [2,3].

Talmor et al. describe a series of 7 cases of abdominal viscera perforation from 1983 to 1995 and showed a mortality rate of 50% [4]. A French review presents 19 cases of incidental perforations between 2001 to 2012, of which three were fatal [5]. Lehnhardt et al. review on liposuction complications in Germany from 1998 to 2003 reports 2275 patients with 73 cases of major complications (23 deaths), of them 10 were bowel perforations (3 deaths) [6].

Lumbar hernia is an uncommon defect of the posterior abdominal wall, that represents less than 1% of all abdominal wall hernias [3,7]. Instead, incisional lumbar hernias can complicate 7% of retroperitoneal surgical approaches [8].

True lumbar incisional hernias have to be distinguished from abdominal wall musculature atrophy caused by division of the lower thoracic nerves can complicate flank incisions.

Patients who develop an uncomfortable and cosmetically displeasing flank bulge resulting from transversus abdominis and oblique muscles laxity often require correction of the defect. Because no wall defect exists, this is not to be considered a true hernia and surgical correction for cosmetic reasons is usually difficult and unsatisfactory [[8], [9], [10], [11], [12]]. Wall muscle plication and interposition of synthetic mesh prosthesis are both conventional methods to correct such deformity.

Aim of this case report is to highlight a rare but potentially lethal complication, that can occur after a very frequent plastic and aesthetic surgical procedure.

## 2 Case presentation

A caucasian, 69 y.o., nonsmoker and not consuming alcohol woman referred to our outpatient office seeking for an abdominal profile reshaping procedure. The patient suffered from hypertension and gastroesophageal reflux, both drug-treated. Her BMI was 25,6 and the anesthesiologist assigned her ASA II score.

**Fig. 1:**
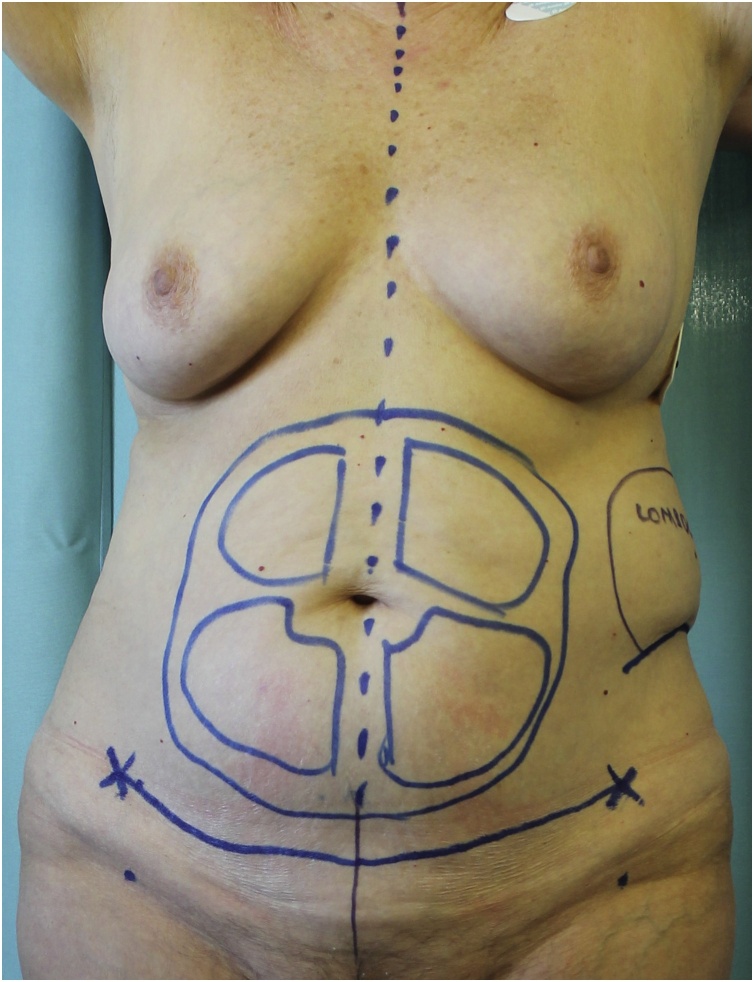
Anterior view with preoperative markings.

**Fig. 2:**
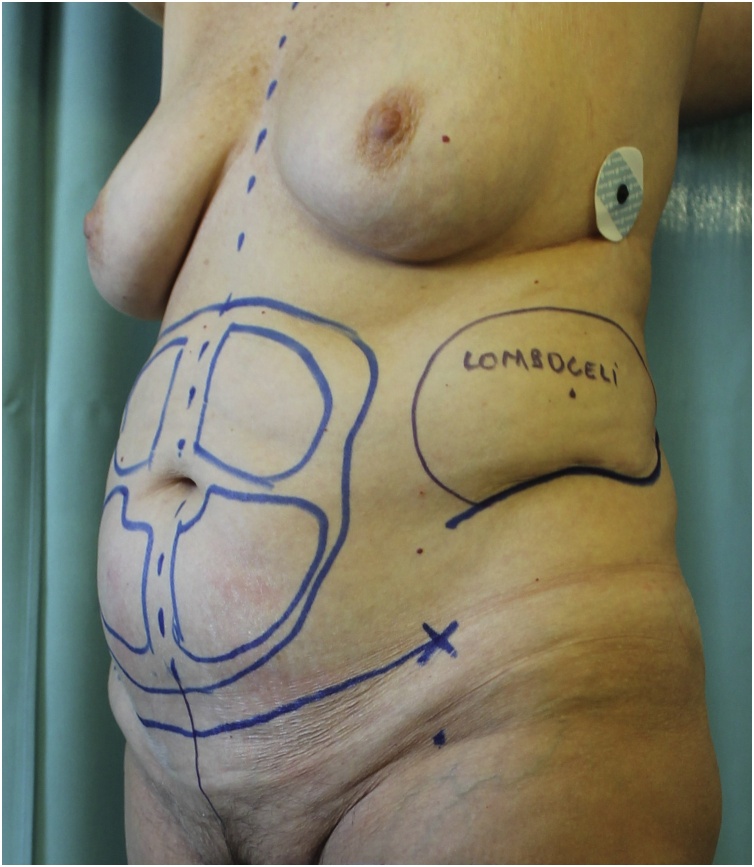
Anterolateral view showing the left flank bulge and the preexisting scar.

**Fig. 3:**
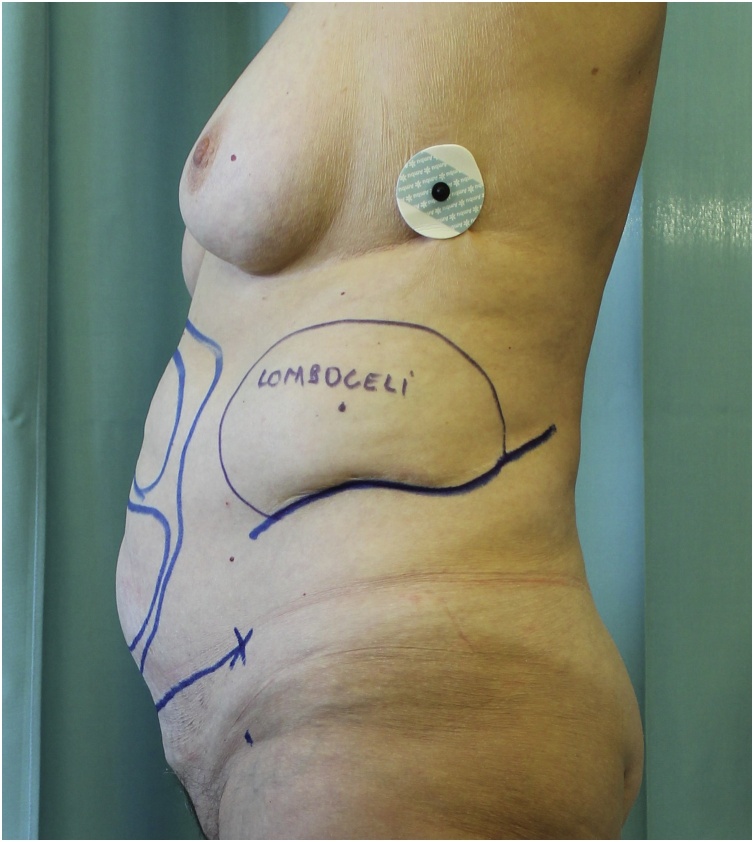
Lateral view of the flank bulge and the preexisting scar (blue line).

The patient presented an 18-cm long linear scar on the left lateral side of her abdomen due to open kidney surgery performed 30 years before for renal stones removal and she did not complain any abdominal symptom at the time of the visit. Thus, trunk contour was characterized by both a bulge deformity of the left side and moderate subcutaneous adipose tissue excess with skin laxity of the inferior abdomen (Fig. 1, Fig. 2, Fig. 3).

At the ultrasound investigation, the bulge revealed a portion of 0,7 cm of relaxed fascia underlying the existing scar with protrusion of fatty tissue through the gap, without any sign of intestinal loops involvement.

The patient was scheduled for flank bulge repair, liposuction and short scar abdominoplasty, which did not include umbilical transposition or recti abdominis muscles plication.

Surgery was performed by a senior consultant specialist of our clinic with the patient under general anesthesia and placed on her right lateral decubitus with a sandbag gel positioner under her right flank. Through a direct skin incision traced along the existing scar we exposed the left bulge: no true adipose tissue hernia was encountered, but only abdominal wall deformity with absence of proper muscle fascia.

A double plication of the left external oblique muscle was performed, after drug-induced muscle relaxation. Turned the patient in supine decubitus we focused on the lower abdomen performing two stab incisions medially to anterior superior iliac spines to infiltrate 800cc of tumescent solution [13]. Then liposuction was performed using a 4 mm blunt cannula and about 800cc of clear adipose tissue were removed without bleeding.

A short scar abdominoplasty completed the procedure, removing conventional skin and subcutaneous flap from the hypogastric area, any sign of muscle wall damage was noticed.

Overall, surgery time took 95 min, antibiotic and antithrombotic prophylaxis therapies were administered.

Day 1# post-op was characterized by vital parameters and laboratory values in normal range, pain 5 on a scale of 10, slight nausea and lack of appetite.

In day 2# post-op a slight rise of body temperature (37.3 °C) occurred, associated with vomiting, skin pallor and gasping (sO2 90%), heart rate was 105. Physical evaluation of the abdomen evoked mild pain, without a positive Blumberg’s sign, neither skin alterations nor inferior limb edema was present. Intestinal transit of gas or solids was absent.

Abdominal radiography revealed free intraperitoneal air in the right hypochondriac region associated with a minor expansion of the pulmonary basal region on the same side. CT-scan confirmed the presence of free air in the abdomen, ascending colon alterations and showed fluids in the perihepatic area and in the small pelvis.

An emergency laparotomy was performed after investigation by exploratory laparoscopy and fecal peritonitis was intraoperatively diagnosed. A portion of ileal loop about 10 cm long injured by multiple perforations was found located at 50 cm from the ileocecal valve. The segment was resected, and an ileo-ileal anastomosis was performed.

**Fig. 4:**
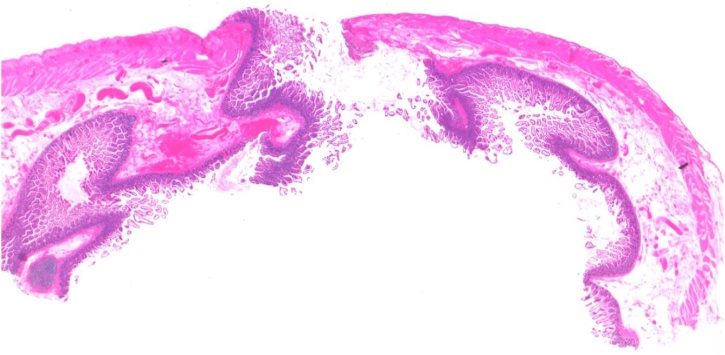
Histopathological image of the resected intestinal loop after haematoxylin-eosin stain: multiple perforations of 2 mm diameter without inflammatory signs have been found along the 10cm long segment.

**Fig. 5:**
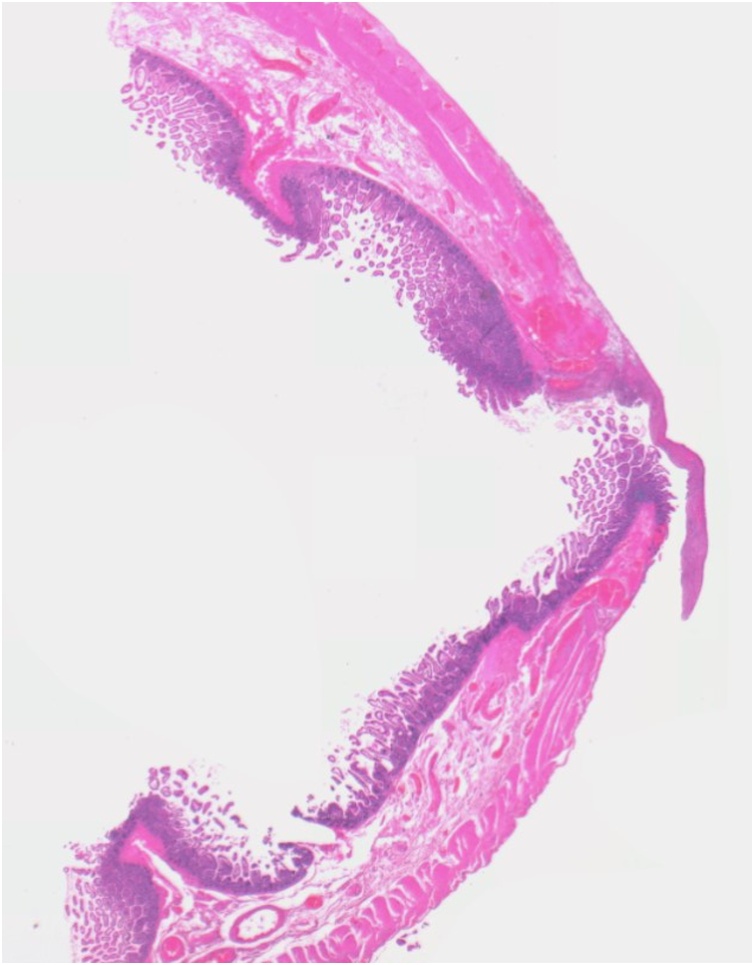
Histopathological image of the resected intestinal loop after haematoxylin-eosin stain: one of the multiple perforations of 2 mm diameter.

The histological examination of the 10 cm long resected loop showed multiple perforations of 2 mm diameter without inflammatory signs (Figs. 4 and 5).

The postoperative 7 days showed to be regular, gradually the patient started eating until the resolution of postoperative paralytic ileus and a complete canalization was reached.

The patient was discharged on the 14th day of hospitalization provided with a diet schedule, drug therapy, and advised to wear a girdle for 3 months. Follow up didn’t show any sign of further complication.

This case report has been reported in line with the SCARE criteria [14].

## 3 Discussion

Multiple surgical procedures are often used in body contouring to obtain a satisfying result. Combined procedures are worldwide accepted in plastic surgery, but data about the increased risk of complications is not unambiguous. Overall, the increased risk of combined procedures is due to the operative time taking more than 3 h [15].

A questionnaire to board-certified members of ASAPS showed a significant increase of complications when liposuction was combined with other procedures [16]. On the other hand, when lipoplasty was combined with abdominoplasty, with or without other procedures, the mortality rate was lower than the mortality rate for abdominoplasty alone (0.0305% vs 0.0414%) [16,17].

Practice Advisory on Liposuction of 2003 recommends limiting liposuction aspiration volumes in other to safely perform combination with additional plastic surgery procedures [18].

In our case liposuction, short scar abdominoplasty and flank reshaping were all considered essential steps to effectively correct patient’s trunk deformities.

Overall surgery time was 95 min and the total aspirated volume was 800cc of clear adipose tissue, according to the guidelines.

In literature cases of incidental bowel perforation are described, even with minimum lesions.

In the current case, multiple point-like wounds of 2 mm diameter were detected by pathologists.

An accredited hypothesis is that liposuction cannula was responsible for these perforations even if the vacuum-assisted cannula had a blunt tip and larger diameter (4 mm). This finding can be explained by bowel wall developing a certain degree of collapse after perforation.

Another interpretation could be that the smaller infiltration cannula (2 mm diameter), used before fat aspiration, has caused the perforations.

The absence of inflammatory tissue reaction around the holes validated the hypothesis of an external trauma instead of a chronic-stress related damage.

Along abdominal wall areas of different resistance to external trauma coexist, among them, the supraumbilical area is accredited to be the most susceptible one [19]. For that reason, some authors suggest that infiltration and liposuction must not be performed with abdomen extended and preferably not performed by the assistant surgeon [5].

In the current case, patient position was supine during aspiration, moreover, there was probably an increased endoabdominal pressure, caused by the flank pseudohernia correction previously performed. Flank correction before abdominal wall procedures were planned to reduce patient position changes and overall surgery time.

The muscles wall plication needed drug-induced myorelaxation, the half-life of curare drug can vary depending on patient weight, age, liver cytochrome activity. One possible scenario is that at the time of liposuction the abdominal wall muscles were still deeply relaxed, and because of this more susceptible to penetrating trauma, even using a blunt tip cannula.

In literature, a similar case of perforation of lumbar hernias is described, but in that case they were unknown and containing intestinal loops [3]. Our patient did not present an anterior abdominal hernia containing bowel, it was a lateral muscles wall bulging, that could not have been damaged during liposuction procedure.

Previous studies described also spontaneous free perforation of the small intestine, however the postoperative course was slower and less painful than in our case [20].

After the body contouring, we didn’t discharge the patient hastily and we monitored her constantly in the postoperative time. Firstly, perforation symptoms were confused with gastroesophageal reflux syndrome, that was reported in patient’s medical history. The early diagnosis allowed the more conservative treatment with bowel anastomosis, avoiding temporary colostomy, and a further operation

If an abdominal perforation is suspected, a chest/abdominal x-ray and abdominal-pelvic CT-scan should be immediately requested. Treatment should be prompt, through laparoscopic or open laparotomy, as well as management of perforations according to their location, size and time of evolution [21].

## 4 Conclusion

Liposuction is a safe procedure if properly performed, but intraoperative changes of patient decubitus, increased endoabdominal pressure and trunk extension could determine a higher risk of iatrogenic damage. Combined procedures in trunk contouring surgery are widely accepted, but the sequence of the procedures could be itself crucial to determine adverse events.

In case of symptomatic intestinal transit alteration after abdominal liposuction it is mandatory to rule out the possibility of a rare but life-threatening complication such as iatrogenic bowel perforation.

Careful monitoring of postoperative clinical course and timely use of radiologic exams are of paramount importance to avoid dramatic consequences.

To our knowledge, this is the first case report of an accidental visceral perforation during a combined procedure of flank bulging correction and abdominal liposuction.

## Author note

In University Hospital of Padua, Italy, Caterina Gardener, MD, is a Plastic Surgeon; laura Pandis, MD, is a Plastic Surgeon; Martina Grigatti, MD, is a Plastic Surgeon; Vincenzo Vindigni, PhD, is a Plastic Surgeon; Franco Bassetto is a Plastic Surgeon and Tito Brambullo, MD, is a Plastic Surgeon. The authors have disclosed no financial relationships related to this article.

## Declaration of Competing Interest

No conflict of interest.

## Sources of funding

No source of funding.

## Ethical approval

It is a retrospective case report and no ethical approval was required.

## Consent

Written informed consent was obtained from the patient for publication of this case report and accompanying images.

## Authors contribution

Caterina Gardener: data collection, literature search, writing and editing of the article.

Laura Pandis: literature search, interpretation and editing of the article.

Martina Grigatti: literature search and data collection.

Vincenzo Vindigni: supervision and contribuition to the submission process.

Franco Bassetto: study concept and validation of the article.

Tito Brambullo: conceptualization, supervision, review and editing of the article.

All authors participated in the design of the case report, read and approved the final manuscript.

## Registration of research studies

Article submitted was a case report and therefore not applicable.

## Guarantor

Primary Surgeon – Prof. Franco Bassetto.

## Provenance and peer review

Not commissioned, externally peer-reviewed.
